# Associations of Social Jetlag With Depression and Anxiety in Adolescents and Young People: A Systematic Review and Meta‐Analysis

**DOI:** 10.1155/da/5542425

**Published:** 2026-01-16

**Authors:** Yi-An Lu, Pei-Shan Tsai

**Affiliations:** ^1^ School of Nursing, College of Nursing, Taipei Medical University, Taipei, Taiwan, tmu.edu.tw; ^2^ Department of Nursing and Research Center in Nursing Clinical Practice, Wan Fang Hospital, Taipei Medical University, Taipei, Taiwan, tmu.edu.tw; ^3^ Research Center of Sleep Medicine, Taipei Medical University Hospital, Taipei, Taiwan, tmuh.org.tw

**Keywords:** adolescents, anxiety, depression, social jetlag, young people

## Abstract

**Background:**

Adolescence and young age are pivotal periods for the emergence of mental health difficulties, marked by major changes in sleep patterns, including an increase in social jetlag (SJL). SJL is often associated with inadequate sleep, shortened sleep, and greater daytime sleepiness, which could potentially lead to mental health problems. This review and meta‐analysis examined the relationships between SJL, depression, and anxiety in this population.

**Methods:**

A systematic search of CINAHL, Embase, PsycINFO, PubMed, and Web of Science identified 14 studies (164,529 participants) examining the association of SJL with depression and anxiety. Pooled associations were calculated using Fisher’s *z* values within a random‐effects model. We assessed heterogeneity with the *I* ^2^ statistic and conducted subgroup and meta‐regression analyses to identify possible sources. The robustness of the results was evaluated through sensitivity analyses, and Egger’s test was applied to examine the possibility of publication bias. The certainty of evidence was assessed using the Grading of Recommendations, Assessment, Development, and Evaluation.

**Results:**

SJL was significantly and positively associated with depression (Fisher’s *z* = 0.27, 95% confidence interval [CI] = 0.16–0.38) and anxiety (Fisher’s *z* = 0.21, 95% CI = 0.12–0.29). Social jetlag of 1–2 h and >2 h was significantly associated with increased odds of depression, with odds ratios of 1.12 (95% CI = 1.05–1.20) and 1.87 (95% CI = 1.73–2.02), respectively.

**Conclusion:**

This review’s findings demonstrate a possible association between SJL and increased odds of depression and anxiety among adolescents and young people, highlighting the importance of addressing sleep–wake misalignment in this population. However, as the certainty of evidence was rated as very low, the results should be interpreted with caution.

## 1. Introduction

The World Health Organization defines adolescence as 10–19 years of age and youth as the 15–24 year age range; collectively, these two groups are referred to as young people [1]. Mental health conditions often emerge in this period [2, 3].

Sleep is a process that results from a complex interplay between external social and environmental cues and internal factors, such as neural networks, hormones, and neurotransmitters [4]. Adolescents and young people experience major changes in their sleep patterns, with their sleep–wake cycle becoming increasingly irregular and delayed. Their sleep pattern is characterized by later bedtimes on both weekdays and weekends, with wake times delayed on weekends and advanced on school days, leading to rhythm desynchronization [5–7]. These changes reduce sleep hours and may lead to misaligned and insufficient sleep. During adolescence, hormonal changes can disrupt sleep patterns, with these physiological shifts altering the endogenous rhythm period and further influencing sleep quality and timing [8, 9]. These changes potentially steer adolescents’ and young people’s circadian preference toward an evening chronotype [6, 10, 11]. This shift resembles the effects of jetlag, leading to social jetlag (SJL), a desynchronization between biological rhythms governed by the internal clock and the external demands of daily routines such as school or work. SJL is typically measured as the time difference between mid‐sleep on free days (MSF) and on workdays (MSW) [12]. Adolescents and young people tend to experience severe SJL due to the constraints of early school start times and lifestyle factors [13].

Evidence suggests that the link between sleep and mental health is intricate and multifaceted. Psychological conditions may disrupt sleep duration and quality, and inadequate sleep can increase susceptibility to mental health problems [14]. Studies indicate that individuals with high SJL are at an increased risk of negative affectivity, including depression, anxiety, anger, inattention, and impaired academic performance [15–17]. Depression is particularly prevalent among students with a late chronotype [18, 19].

Although many studies have explored the relationship between sleep and mental health in adolescents, this relationship remains incompletely understood [20]. A previous systematic review by Henderson et al. [21] on the relationship between SJL and mental health in young people concluded that the evidence remained uncertain, as earlier studies lacked consensus and did not employ a standardized approach to calculating the associations of SJL with depression and anxiety. However, that review included both clinical (i.e., participants with major depressive disorder) and non‐clinical populations, as well as both employed and unemployed individuals. This broad scope made it difficult to isolate the effects of SJL in otherwise healthy adolescents and limited its relevance specifically to adolescents and young people. Additionally, the study sample in that review was restricted to populations from Europe, the United States, and South America, with a total sample size of ~9000, which hindered the generalizability of its findings.

To this end, we aimed to conduct a systematic review and meta‐analysis, incorporating a larger and more geographically diverse dataset by expanding the sample to include participants from Asia, those from Europe, the United States, and South America. Furthermore, we addressed methodological inconsistencies in previous research by applying robust meta‐analytic techniques to enhance the validity of the findings. In the present systematic review and meta‐analysis, we investigated the association between SJL and measures of depression and anxiety by analyzing observational studies from diverse geographic regions. Our study specifically focuses on individuals aged 10–24 years. By utilizing standardized assessment tools to improve comparability and addressing methodological inconsistencies, our study provides evidence for more reliable estimation of the association between SJL and depression and anxiety.

## 2. Methods

The study adhered to the Preferred Reporting Items for Systematic Reviews and Meta‐Analyses (PRISMA) guidelines [22]. The study protocol was registered on PROSPERO (Registration No. CRD 42024627930).

### 2.1. Data Sources and Search Strategy

We systematically searched the following databases for studies published prior to December 15, 2024: CINAHL, Embase, PsycINFO, PubMed, and Web of Science. The search strategy included the following keywords: (social jet lag) OR (jetlag) OR (jet lag) OR (jet‐lag) OR (sleep debt) AND (anxiety) OR (depression) OR (mental health) OR (psychological) AND (adolescent) OR (young people) OR (young adulthood). Details regarding the search strategy are provided in Supporting Information: Table S1.

### 2.2. Inclusion and Exclusion Criteria

Studies were considered eligible for inclusion if they (1) involved participants between 10 and 24 years of age; (2) adopted a cross‐sectional, cohort, prospective, or retrospective design; and (3) evaluated depression or anxiety in association with SJL. No language restrictions were applied to any study. Studies published as conference abstracts, reviews, or case reports and studies with an experimental design were excluded. Studies lacking appropriate outcome measures or original data were also excluded.

### 2.3. Study Selection and Data Extraction

References were managed and duplicates removed using EndNote 20 software. To select studies, two investigators (Y.A.L. and P.S.T.) independently reviewed the titles, abstracts, and full‐text articles to assess eligibility, with any discrepancies resolved through discussion. If consensus could not be achieved, a third reviewer was involved to provide adjudication.

We compiled data from all eligible studies into standardized tables, capturing study characteristics (e.g., first author, year of publication, country, study design, sample size, participants’ age, and female participant ratio), mental health outcome measures, and key findings regarding the association of SJL with anxiety and depression were extracted.

### 2.4. Evaluation of Study Quality

We used the Appraisal Tool for Cross‐Sectional Studies (AXIS) [23] to evaluate the methodological quality of the included studies. This tool offers a comprehensive framework that consists of 20 questions focusing on three main areas: design quality (seven items), reporting quality (seven items), and potential bias (six items). Each question is answered with either “Yes,” “No,” or “Do not know” [24]. Study quality was evaluated using 20 criteria, with scores ≥16 indicating high quality [25]. In this study, assessments were independently performed by two reviewers, with a third reviewer consulted to resolve any inconsistencies.

### 2.5. Evaluation of Evidence Certainty

The certainty of the evidence was appraised using the GRADE (Grading of Recommendations, Assessment, Development, and Evaluation) framework and classified as high, moderate, low, or very low. Ratings for nonrandomized or observational studies initially started at low and were adjusted on the basis of factors such as risk of bias, inconsistency, indirectness, imprecision in study results, and publication bias [26].

### 2.6. Data Synthesis and Statistical Analysis

To evaluate the association of SJL with depression and anxiety, various indices reported in the included studies were converted into a common index, namely a correlation coefficient (*r*), by following the methods proposed by Borenstein et al. [27]. For studies reporting regression analyses, standardized beta coefficients were extracted and converted into correlation coefficients, as described by Bring [28]. Correlation coefficients were subsequently standardized into Fisher’s *z* values using R software (version 4.3.1). Fisher’s *z* values and their corresponding standard errors were calculated using sample size (*N*) and correlation coefficients, as outlined in [29].

Meta‐analyses were conducted when at least two studies addressed the same research outcome. Random‐effects pairwise meta‐analyses were used to synthesize pooled Fisher’s *z* and odds ratio (OR) values, along with their corresponding 95% confidence intervals (CIs), using Review Manager software (RevMan, version 5.3). Fisher’s *z* values were applied when pooling studies that reported correlations or regression‐based standardized estimates, allowing us to synthesize continuous associations between SJL and psychological outcomes. In contrast, for studies that categorized SJL into discrete groups and reported risk estimates, we directly pooled ORs to evaluate group differences. Effect sizes were interpreted on the basis of Cohen’s criteria and classified as small (0.10–0.30), medium (0.30–0.50), or large (>0.5) [30]. Heterogeneity was assessed using the *I* ^2^ statistic and categorized as low (<25%), moderate (26%–50%), or high (>50%) [31].

To test the robustness of the findings, we performed sensitivity analyses excluding studies that were not classified as high quality. Studies were deemed not to be high quality if they had an AXIS score of <16. The effect of excluding these studies on the overall results was evaluated.

Subgroup analyses were conducted to identify potential contributors to variability in cases of substantial heterogeneity (*I* ^2^ ≥ 50%). For depression, subgroup analyses were based on (1) the cutoff values for determining SJL, categorized into four groups: SJL < 1 h, 1–2 h, <2 h, and SJL ≥ 2 h; (2) the type of questionnaire used for measurement; and (3) the type of student population examined. For anxiety, subgroup analyses were conducted according to different SJL cutoff values. We also applied meta‐regression for continuous variables to explore potential sources of variation. Meta‐regressions based on age and female representation were performed when sufficient data were available.

Assessment of publication bias was conducted using Egger’s test and further evaluated through funnel plot visualization for both depression and anxiety. The trim‐and‐fill method was employed to address publication bias by imputing potentially missing studies and correcting funnel plot asymmetries, using R software (version 4.3.1) [32].

## 3. Results

### 3.1. Study Selection

**Figure 1:**
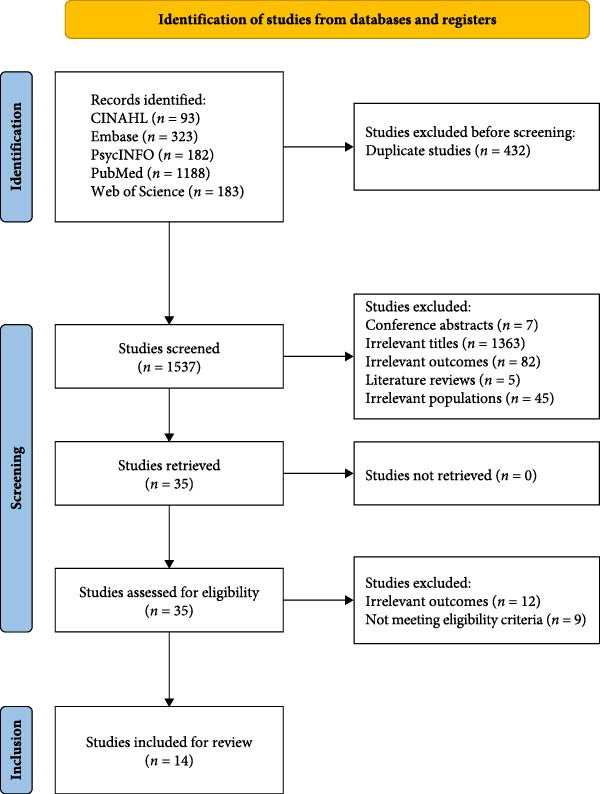
PRISMA flow diagram of study selection.

Database searches identified 1969 studies, of which 432 duplicates were excluded. After titles and abstracts were screened, another 1502 studies were excluded, yielding 35 studies selected for full‐text review, of which 21 were deemed ineligible according to the inclusion criteria and were subsequently excluded. Ultimately, 14 studies were included for analysis [33–46]. Figure 1 illustrates the process of study selection.

### 3.2. Study Characteristics

Overall, the included studies involved 164,529 adolescents and young people, with a mean age of 13.42 years. Most studies originated in Asia (*n* = 6), followed by Europe (*n* = 4), the United States (*n* = 3), and South America (*n* = 1). The number of participants per study ranged from 65 to 37,871, with a mean of 11,752.

SJL was primarily evaluated using self‐report questionnaires, such as the Munich Chronotype Questionnaire. In the majority of studies, SJL was calculated on the basis of MSW and MSF by using the following formula for SJL in hours: |MSF −  MSW|. To refine the analysis and account for sleep debt, one study also calculated sleep‐corrected SJL (SJLsc) by using the following formula: |MSFsc −  MSWsc| [38]. Additionally, one study employed objective parametric measures of sleep duration by calculating average sleep onset and sleep conclusion in school and free nights to determine the average sleep midpoint for evaluating SJL [39].

Depression was evaluated using the Beck Depression Inventory [47, 48], the Center for Epidemiological Studies Depression Scale [49, 50], the Depression Self‐Rating Scale for Children [51], the Patient Health Questionnaire [52], and the Seasonal Pattern Assessment Questionnaire [53, 54]. Anxiety was evaluated using the Brief Symptom Inventory [55], the Generalized Anxiety Disorder Scale [56, 57], and the State–Trait Anxiety Inventory [58]. Additionally, both depression and anxiety were evaluated using the Depression, Anxiety, and Stress Scale [59] and the Patient‐Reported Outcomes Measurement Information System [60]. Eight studies focused on depression [33, 34, 36, 37, 40, 43, 44, 46], two studies focused on anxiety [35, 41], and four studies focused on both depression and anxiety [38, 39, 42, 45]. Table 1 presents the studies included in the review. The measurement tools used in the included studies were validated and widely applied in research, demonstrating robust psychometric properties, including high internal consistency (Cronbach’s *α* > 0.80), reliability, and validity.

**Table 1:** Summary of included studies.

No.	Author (year), country	Participants	Study design	Participants			SJL (h or min)	Outcome measures	Findings
				Sample size	Mean ± SD (age range in years)	Female (%)			
1	Borisenkov et al. (2015), Russia	Secondary and high school students	Cross‐sectional	3435	14.8 ± 2.6(10–20)	1918 (55.8)	NA	Depression (SPAQ)	SJL had a significant effect on depression in females, but not in males
2	de Souza et al. (2014), Brazil	Adolescent students	Cross‐sectional	351	14.70 ± 1.86 (12–21)	247 (70.4)	<2 h ≥2 h	Depression (BDI)	SJL was not significantly associated with depression
3	Díaz‐Morales (2016), Spain	Adolescent high school students	Cross‐sectional	1406	13.95 ± 1.69 (12–16)	716 (50.9)	NA	Anxiety (STAI)	SJL was not significantly associated with anxiety
4	Jang et al. (2021), Korea	Nursing students	Cross‐sectional	304	20.56 ± 1.70 (19–21)	304 (100)	NA	Depression (CES‐D)	SJL was significantly associated with depression
5	Jang and Lee (2023), Korea	Nursing students	Cross‐sectional	198	21.91 ± 1.49 (21–23)	174 (87.9)	NA	Depression (CES‐D)	SJL was significantly associated with depression
6	Li et al. (2024), China	Adolescent students	Cross‐sectional	106,979	13.0 ± 1.8 (10–18)	481,683 (45.5)	<1 h 1 and 2 h ≥2 h	Depression (PHQ‐2) Anxiety (GAD‐2)	Increased SJL and SJLsc were significantly associated with an increased risk of depression and anxiety
7	Magnusdottir et al. (2024), Iceland	Adolescent students	Cohort	65	17.3 ± 0.08 (16–19)	41 (63.0)	NA	Depression (BDI‐II) Anxiety (GAD‐7)	SJL was significantly associated with depression, but not with anxiety
8	Mathew et al. (2019)^a^ USA	Adolescents	Cross‐sectional	3058	15.59 ± 0.77	1478 (48.3)	NA	Depression (CES‐D)	SJL had a significant effect on depression
9	Mathew et al. (2019)^b^ USA	Adolescents	Cross‐sectional	3097	15.59 ± 0.77	1494 (48.2)	<2 h >2 and ≤4 h >4 h	Anxiety (BSI‐18)	SJL had a significant effect on anxiety
10	Sheaves et al. (2016), UK	Undergraduate/postgraduate students	Cross‐sectional	1403	21.34 ± 2.23 (20–23)	780 (55.6)	NA	Depression (DASS‐21) Anxiety (DASS‐21)	SJL was not significantly associated with depression or anxiety
11	Tamura and Okamura (2023)^a^ Japan	Adolescent junior high school students	Cohort	427	13.3 ± 0.6 (13–14)	193 (45.2)	<1 h ≥1 h ≥2 h	Depression (DSRS‐C)	Increased SJL was significantly associated with an increased risk of depression
12	Tamura and Okamura (2023)^b^ Japan	Adolescent students	Cross‐sectional	1493	13.0 ± 0.9 (12–15)	783 (52.4)	<0 h 0 and <1 h 1 and <2 h ≥2 h	Depression (DSRS‐C)	Increased SJL was significantly associated with an increased risk of depression in females, but not in males
13	Wong et al. (2024), USA	Adolescent middle and high school students	Cross‐sectional	4442	15.35 ± 3.71 (13–18)	2305 (51.9)	<2 h ≥2 h	Depression (PROMIS) Anxiety (PROMIS)	SJL was significantly associated with depression and anxiety
14	Zhang et al. (2023), China	Adolescent high school students	Cross‐sectional	37,871	13.50 ± 0.76	17,417 (46.0)	<1 h 1 to 2 h ≥2 h	Depression (PHQ‐9)	Increased SJL was significantly associated with an increased risk of depression

*Note:* Superscript letters a and b indicate different articles published by the same authors in the same year.

Abbreviations: BDI, Beck Depression Inventory; BSI, Brief Symptom Inventory; CES‐D, Center for Epidemiological Studies Depression Scale; DASS, Depression, Anxiety, and Stress Scale; DSRS‐C, Depression Self‐Rating Scale for Children; GAD, Generalized Anxiety Disorder Scale; NA, not applicable; PHQ, Patient Health Questionnaire; PROMIS, Patient‐Reported Outcomes Measurement Information System; SJL, social jet lag; SJLsc, sleep‐corrected social jet lag; SPAQ, Seasonal Pattern Assessment Questionnaire; STAI, State–Trait Anxiety Inventory.

### 3.3. Results of Quality Assessments

Study quality scores ranged from 13 to 18; however, six studies failed to achieve the ≥16 cutoff for classification as high quality. Reporting quality criteria were the most consistently satisfied among all domains (range = 5–7, mean = 6.57). All included studies specified their research aims and objectives, and the majority of them provided detailed descriptions of their methodologies, measures, and study populations. However, participant characteristics were not clearly defined in four studies [33–35, 42], and study limitations were not outlined in one study [35], contributing to an unclear research focus in these studies.

Study design criteria were met to a limited extent (range = 4–7, mean = 5.5). The use of cross‐sectional designs was suitable for the objectives of all studies, with outcome variables largely consistent with those aims. Additionally, ethical approval was obtained in all of the selected studies. However, five studies [34, 36, 37, 39, 40] provided limited descriptions of the sampling procedures, and eight studies [35, 37, 38, 42–46] raised potential concerns regarding the interpretation of the results due to funding sources.

**Figure 2:**
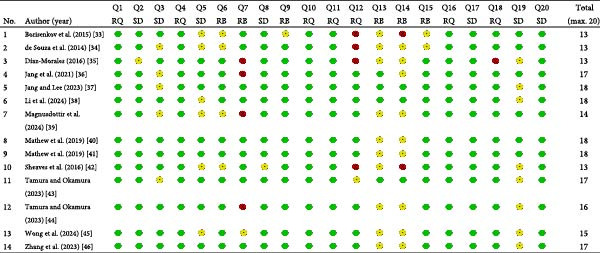
AXIS quality assessment ratings. AXIS, appraisal tool for cross‐sectional studies. Assessment domain: RQ, reporting quality; SD, study design; RB, risk of bias. Ratings: 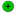 = yes (criteria met); 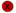 = no (criteria not met); 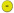 = do not know (criteria partially met).

Questions related to the risk of bias were inadequately addressed in the majority of studies (range = 1–6, mean = 3.5). Limited information was provided on nonresponders, recruitment strategies, and concerns regarding nonresponse bias. Specifically, four studies did not implement measures to address or categorize nonresponse bias [35, 36, 39, 44], and two studies did not provide a description of nonresponders [33, 42]. Figure 2 presents the details of the risk‐of‐bias assessment.

### 3.4. Association Between SJL and Depression

A total of 17 effect estimates obtained from 12 studies were included in the meta‐analysis. The results indicated that SJL was significantly associated with depression when analyzed as a continuous variable based on psychometric scale scores (Fisher’s *z* = 0.27, 95% CI = 0.16–0.38, *I* ^2^ = 100%, *p* < 0.001; Table 2). Additionally, six estimates obtained from four studies were pooled to determine the odds of depression between different SJL definitions when analyzed as a categorical variable. Compared with SJL for less than 1 h, SJL for 1–2 h was significantly associated with increased odds of depression (OR = 1.12, 95% CI = 1.05–1.20, *I* ^2^ = 0%, *p* = 0.001; Table 3A). Similarly, compared with SJL for less than 1 h, SJL for more than 2 h demonstrated a significant associattion with higher odds of depression (OR = 1.87, 95% CI = 1.73–2.02, *I* ^2^ = 0%, *p*  < 0.001; Table 3B). However, no statistically significant association was detected between SJL for less than 2 h and SJL for 2 or more hours (OR = 1.03, 95% CI = 0.63–1.67, *I* ^2^ = 55%, *p* = 0.92; Table 3C).

**Table 2:** Association between SJL and depression.

Study	Fisher’s *z*	SE	Weight	Fisher’s *z* IV, random, 95% CI	Fisher’s *z* IV, random, 95% CI
Borisenkov et al. (2015)	0.333	0.017	5.7%	0.33 (0.30, 0.37)	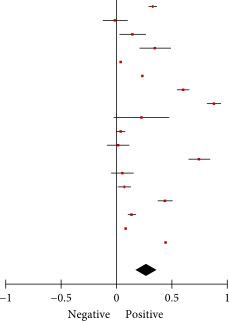
de Souza et al. (2014), SJL ≥ 2 h	−0.007	0.054	5.5%	−0.01 (−0.11, 0.10)	
Jang et al. (2021)	0.151	0.058	5.4%	0.15 (0.04, 0.26)	
Jang and Lee (2023)	0.354	0.072	5.3%	0.35 (0.21,0.50)	
Li et al. (2024), SJL = 1 and 2 h	0.043	0.003	5.8%	0.04 (0.04, 0.05)	
Li et al. (2024), SJLsc = 1 and 2 h	0.24	0.003	5.8%	0.24 (0.23, 0.25)	
Li et al. (2024), SJL ≥ 2 h	0.604	0.026	5.7%	0.60 (0.55, 0.65)	
Li et al. (2024), SJLsc ≥ 2 h	0.884	0.031	5.7%	0.88 (0.82, 0.94)	
Magnusdottir et al. (2024)	0.232	0.127	4.4%	0.23 (−0.020, 0.48)	
Mathew et al. (2019)^a^	0.04	0.018	5.7%	0.04 (0.00, 0.08)	
Sheaves et al. (2016)	0.02	0.05	5.5%	0.02 (−0.08, 0.12)	
Tamura and Okamura (2023)^a^ SJL = 1 and 2 h	0.752	0.049	5.5%	0.75 (0.66, 0.85)	
Tamura and Okamura (2023)^a^ SJL ≥ 2 h	0.057	0.049	5.5%	0.06 (−0.04, 0.15)	
Tamura and Okamura (2023)^b^ SJL = 1 and 2 h	0.077	0.029	5.7%	0.08 (0.02, 0.13)	
Tamura and Okamura (2023)^b^ SJL ≥ 2 h	0.445	0.032	5.7%	0.45 (0.38, 0.51)	
Wong et al. (2024), SJL ≥ 2 h	0.144	0.016	5.7%	0.14 (0.11, 0.18)	
Zhang et al. (2023), SJL = 1 and 2 h	0.085	0.001	5.8%	0.09 (0.08, 0.09)	
Zhang et al. (2023), SJL ≥ 2 h	0.449	0.001	5.8%	0.45 (0.45, 0.45)	
Total (95% CI)	—	—	100%	0.27 (0.16, 0.38)	
Heterogeneity: *τ* ^2^ = 0.05, *χ* ^2^ = 72600.98, df = 19 (*p* < 0.00001), *I* ^2^ = 100%					
Test for overall effect: *Z* = 4.88 (*p* < 0.00001)					

*Note:* Superscript letters a and b indicate different articles published by the same authors in the same year.

Abbreviations: SJL, social jetlag; SJLsc, sleep‐corrected social jetlag.

**Table 3:** Subgroup analyses of risk of depression associated with SJL stratified by SJL definition.

[A] Study	SJL = 1 and 2 h		SJL < 1 h		Weight	OR M–H, random, 95% CI	OR M–H, random, 95% CI
	Events	Total	Events	Total			
Tamura and Okamura (2023)^b^	121	498	156	695	6.7%	1.11 (0.85, 1.45)	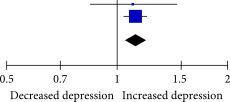
Zhang et al. (2023)	2006	17,234	1329	13,245	93.3%	1.12 (1.04, 1.21)	
Total (95% CI)	—	17,732	—	13,940	100%	1.12 (1.05, 1.20)	
Total events	2127	—	1548	—	—	—	
Heterogeneity: *τ* ^2^ = 0.00, *χ* ^2^ = 0.01, df = 4 (*p* = 0.94), *I* ^2^ = 0%							
Test for overall effect: *Z* = 3.19 (*p* = 0.001)							

**[B] Study**	**SJL > 2 h**		**SJL < 1 h**		**Weight**	**OR** **M–H, random, 95% CI**	**OR** **M–H, random, 95% CI**
	**Events**	**Total**	**Events**	**Total**			

Tamura and Okamura (2023)^b^	105	300	156	695	7.0%	1.86 (1.38, 2.50)	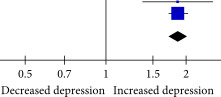
Zhang et al. (2023)	1329	7392	1329	13,245	93.0%	1.87 (1.72, 2.02)	
Total (95% CI)	—	7692	—	13,940	100%	1.87 (1.73, 2.02)	
Total events	1434	—	1497	—	—	—	
Heterogeneity: τ^2^ = 0.00, *χ* ^2^ = 0.00, df = 1 (*p* = 0.98), *I* ^2^ = 0%							
Test for overall effect: *Z* = 15.60 (*p* < 0.00001)							

**[C] Study**	**SJL ≥ 2 h**		**SJL < 2 h**		**Weight**	**OR** **M–H, random, 95% CI**	**OR** **M–H, random, 95% CI**
	**Events**	**Total**	**Events**	**Total**			

de Souza et al. (2014)	15	154	26	197	31.5%	0.71 (0.36, 1.39)	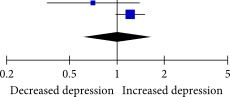
Wong et al. (2024)	1458	1610	2022	2278	68.5%	1.21 (0.98, 1.50)	
Total (95% CI)	—	1764	—	2475	100%	1.03 (0.63, 1.67)	
Total events	1473	—	2048	—	—	—	
Heterogeneity: *τ* ^2^ = 0.08, *χ* ^2^ = 2.22, df = 1 (*p* = 0.14), *I* ^2^ = 55%							
Test for overall effect: *Z* = 0.10 (*p* = 0.92)							

*Note:* Superscript letter b indicates a different article published by the same authors in the same year.

Abbreviation: SJL, social jetlag.

Subgroup analyses were conducted on the basis of (1) cutoff values for determining SJL, (2) the type of questionnaire used for measurement, and (3) student type. Analysis of depression outcomes revealed a significant difference in the association between SJL and depression with different cutoff values used to define SJL (*p* = 0.001). Specifically, a small and positive correlation was observed among individuals with SJL for 1–2 h (Fisher’s *z* = 0.22, 95% CI = 0.14–0.30, *I* ^2^ = 100%, *p* < 0.001), and a medium and positive correlation was observed among individuals with SJL for 2 or more hours (Fisher’s *z* = 0.49, 95% CI = 0.32–0.66, *I* ^2^ = 99%, *p* < 0.001). However, no statistically significant differences were identified between individuals with SJL for more than 2 h and those with SJL for less than 2 h (Supporting Information: Table S2.1A–C). Additionally, no statistically significant difference was identified in the type of questionnaire used for measurement (Supporting Information: Table S2.2A–E). Analysis of student type did not reveal significant differences between groups. However, in college students (aged 19–23 years), the association between SJL and depression was not significant (Supporting Information: Table S2.3A,B).

Meta‐regression analysis revealed that the heterogeneity observed in the association between SJL and depression was not significantly influenced by age or sex (*p* > 0.05, Supporting Information: Table S4). No significant publication bias was detected for depression (*p* = 0.890). Supporting Information: Figure S1A presents a funnel plot of publication bias.

### 3.5. Association Between SJL and Anxiety

After pooling nine effect estimates from six studies, the results indicated a statistically significant association between SJL and anxiety when analyzed as a continuous variable (Fisher’s *z* = 0.21, 95% CI = 0.12–0.29, *I* ^2^ = 99%, *p* < 0.001; Table 4).

**Table 4:** Association between SJL and anxiety.

Study	Fisher’s *z*	SE	Weight	Fisher’s *z* IV, random, 95% CI	Fisher’s *z* IV, random, 95% CI
Díaz‐Morales et al. (2016)	0.310	0.027	12.7%	0.31 (0.26, 0.36)	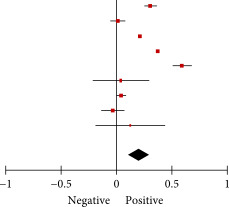
Li et al. (2024), SJL = 1 and 2 h	0.015	0.034	12.4%	0.01 (−0.05, 0.08)	
Li et al. (2024), SJLsc = 1 and 2 h	0.216	0.003	13.3%	0.22 (0.21, 0.22)	
Li et al. (2024), SJL ≥ 2 h	0.381	0.004	13.3%	0.38 (0.37, 0.39)	
Li et al. (2024), SJLsc ≥ 2 h	0.599	0.041	12.0%	0.60 (0.52, 0.68)	
Magnusdottir et al. (2024)	0.045	0.127	6.6%	0.04 (−0.20, 0.29)	
Mathew et al. (2019)^b^	0.05	0.018	13.1%	0.05 (0.01, 0.09)	
Sheaves et al. (2016)	−0.03	0.05	11.5%	−0.03 (−0.13, 0.07)	
Wong et al. (2024), SJL ≥ 2 h	0.129	0.16	5.0%	0.13 (−0.18, 0.44)	
Total (95% CI)	—	—	100%	0.21 (0.12, 0.29)	
Heterogeneity: *τ* ^2^ = 0.02, *χ* ^2^ = 1406.07, df = 8 (*p* < 0.00001), *I* ^2^ = 99%					
Test for overall effect: *Z* = 4.51 (*p* < 0.0001)					

*Note:* Superscript letter b indicates a different article published by the same authors in the same year.

Abbreviations: SJL, social jetlag; SJLsc, sleep‐corrected social jetlag.

Subgroup analyses were conducted on the basis of the cutoff values for determining SJL. Analysis of anxiety outcomes revealed a significant difference in the association between SJL and anxiety with different cutoff values for determining SJL (*p* < 0.001). No statistically significant association was observed between SJL and anxiety in individuals with SJL for 1–2 h (Supporting Information: Table S3A). However, a medium and positive correlation was observed between SJL and anxiety in individuals with SJL for more than 2 h (Fisher’s *z* = 0.49, 95% CI = 0.27–0.70, *I* ^2^ = 96%, *p* < 0.001; Supporting Information: Table S3B). Additionally, a weak and positive correlation was observed between SJL and anxiety in individuals with SJL for more than 2 h (Fisher’s *z* = 0.05, 95% CI = 0.02–0.09, *I* ^2^ = 93%, *p* < 0.001; Supporting Information: Table S3C).

With the exception of the cutoff values for determining SJL, no significant effects of potential moderators considered in the subgroup analyses were observed on the association between SJL and anxiety. The association between SJL and anxiety was significantly influenced by age (*p* = 0.015), but not by sex (Supporting Information: Table S4). No significant publication bias was detected for anxiety (*p* = 0.830). Supporting Information: Figure S1B presents a funnel plot of publication bias.

### 3.6. Sensitivity Analysis

To evaluate the robustness of the meta‐analysis findings, we performed sensitivity analyses removing low‐quality studies (i.e., AXIS score less than 16). The results indicated that the exclusion of five low‐quality studies from the pooled data on depression had a minimal effect on the results (*p* < 0.001). Similarly, a sensitivity analysis excluding data from four lower‐quality studies on anxiety revealed minimal changes in the findings (*p* < 0.001). Supporting Information: Table S5 presents the sensitivity analysis results of the outcomes of interest.

### 3.7. Evidence Certainty

Evidence certainty was evaluated within the GRADE framework. Although depression was a major outcome, its certainty of evidence was rated as “very low” (Supporting Information: Table S6) because of the increased risks of bias, inconsistency, and indirectness. Consequently, the overall evidence regarding the association between SJL and depression remained highly uncertain. Similarly, the evidential certainty related to anxiety was rated as “very low” because of the increased risks of bias, inconsistency, indirectness, and imprecision. Consequently, the overall evidence regarding the association between SJL and anxiety remained highly uncertain (Supporting Information: Table S6).

## 4. Discussion

The findings of this systematic review and meta‐analysis indicate a significant association between SJL and mental health outcomes, with a specific focus on depression and anxiety in adolescents and young people. Although the positive associations of SJL with both depression and anxiety exhibited small effect sizes, the high heterogeneity observed across studies underscored the complexity and multifaceted nature of these associations.

Although heterogeneity was high for both outcomes, this variability is expected when synthesizing observational studies from diverse cultural and developmental contexts. The random‐effects model was therefore used to estimate the overall trend rather than a single common effect. Subgroup and meta‐regression analyses indicated that SJL cutoff definitions and participant age were the major contributors to heterogeneity. The pooled Fisher’s *z* values should thus be interpreted as indicators of general directionality rather than precise effect magnitudes.

In this study, we discovered that SJL is linked to both an increased likelihood and greater severity of depression. This finding is consistent with those of Im et al. [61] and Min et al. [62], who argued that SJL is significantly associated with depressive mood, with depressive symptoms increasing with increasing SJL. These findings suggest that habitual exposure to circadian misalignment during workdays contributes to the development and exacerbation of depression.

Our subgroup analyses revealed significant differences in the associations between various types of students. Research indicates that sleep deprivation and low sleep quality are key risk factors for depression, particularly among adolescents [17]. However, among college students (aged 19–23 years), the association between SJL and depression was not significant, consistent with previous findings showing considerable variability in the impact of SJL across different age groups [21]. This result should be interpreted with caution given the high heterogeneity and the limited number of studies in this subgroup. The absence of a significant association among college students may be explained by differences in lifestyle, greater autonomy, and more flexible daily schedules, which can weaken or obscure the measurable link between SJL and depressive symptoms. Moreover, college students may possess more developed coping mechanisms and reduced sensitivity to circadian misalignment compared with adolescents, who are more vulnerable to the effects of sleep deprivation and poor sleep quality [21]. Collectively, these factors suggest that the non‐significant association observed in this subgroup is likely exploratory and hypothesis‐generating, highlighting the need for further research with larger and more homogeneous samples to confirm whether this pattern persists across age groups.

In our study, we observed that SJL is positively correlated with heightened anxiety severity. This finding is consistent with those of Jung and Lee [63], who demonstrated that the misalignment of sleep patterns is closely linked to anxiety. According to the literature, individuals with SJL typically experience circadian misalignment between their internal biological clock and socially dictated schedules. This misalignment often leads to chronic sleep debt, increased stress, and hormonal fluctuations, particularly in the levels of melatonin and cortisol, which are key regulators of mood and anxiety. These disruptions collectively contribute to increased anxiety levels [64, 65].

Our meta‐regression analyses revealed that age significantly influenced the association between SJL and anxiety. This finding may be attributable to the hormonal changes that occur during different developmental stages. During puberty, adolescents experience fluctuations in the levels of hormones such as cortisol and melatonin, which can amplify the effects of circadian misalignment on anxiety. High cortisol levels, commonly observed in individuals with SJL, are strongly associated with increased anxiety symptoms [65]. By contrast, young adults often benefit from flexible schedules and autonomy over their sleep routines, which may help mitigate the severity of the anxiety symptoms associated with SJL. This flexibility enables young adults to better align their sleep–wake cycles with their internal biological clocks, mitigating the effect of circadian misalignment on mental health [66]. The broad age range (10–24 years) encompassed in this review spans major developmental transitions, from early adolescence to young adulthood. Variations in hormonal regulation, parental supervision, academic demands, social expectations, and employment responsibilities across these stages likely contribute to the heterogeneity observed in our meta‐analytic findings.

Cultural and societal contexts may also play a significant role in shaping both the prevalence and psychological consequences of SJL. For instance, East Asian societies are often characterized by earlier school start times, heavier academic workloads, and longer evening study hours, which can exacerbate sleep restriction and circadian misalignment in adolescents. In contrast, many European countries have implemented later school start times and more flexible daily schedules, potentially mitigating SJL‐related stress. In North and South America, diverse socioeconomic and work patterns may further influence sleep timing and variability [67, 68]. These contextual factors could partly explain the regional variability and heterogeneity observed in our analyses. Future studies directly comparing cultural contexts may help identify specific societal drivers of SJL and clarify whether its mental health impact differs across regions.

This study has several strengths. First, our large sample size offers a broad representation of adolescents and young people across multiple regions, enhancing the generalizability of the results. Second, sensitivity analyses were conducted to test the robustness of the findings, which provided support for their validity. Third, most of the included studies employed standardized definitions and measurement tools for SJL, which enhance the comparability and credibility of depression and anxiety assessments across studies.

Despite these strengths, several limitations should be acknowledged. Approximately 40% of the included studies were rated below the AXIS threshold for high quality, primarily due to incomplete descriptions of sampling procedures and lack of discussion on nonresponse bias. However, all studies used validated psychological instruments and consistent SJL definitions, allowing meaningful synthesis. Sensitivity analyses excluding these lower‐quality studies yielded nearly identical results (Fisher’s *z* = 0.32 for depression; 0.25 for anxiety), confirming the robustness of the associations. Nevertheless, these methodological limitations contributed to downgrading the certainty of evidence to very low in the GRADE assessment, and the pooled estimates should therefore be interpreted with caution.

The included studies comprised 12 cross‐sectional and two cohort designs. Although the limited number of cohort studies precluded a subgroup analysis according to study design, the available longitudinal evidence generally supported the direction of the associations observed in cross‐sectional studies. In Magnusdottir et al. [39], which included a relatively small sample (*n* = 65), SJL was significantly associated with depression but not with anxiety. In contrast, Tamura and Okamura [43] found that greater SJL was prospectively associated with an increased risk of depression in a larger cohort of Japanese adolescents (*n* = 427). Despite these variations, both cohort studies suggested that SJL may precede the onset or worsening of depressive symptoms. However, the small number of prospective studies and limited statistical power restrict the strength of inference. Future research incorporating larger samples and repeated measures of SJL and mental health outcomes is warranted to confirm temporal patterns and potential causal pathways.

Additionally, the reliance on observational study designs limited our ability to establish causal inferences. The use of self‐reported measures for SJL and mental health may have introduced recall bias, potentially affecting the accuracy of the data. Moreover, the variability observed in the definitions and measurement tools used for depression and anxiety across studies may complicate comparisons and the synthesis of results. Although subgroup and meta‐regression analyses identified some sources of variation, the substantial heterogeneity could not be fully accounted for. Finally, the certainty of evidence was rated as “very low” for the two associations of interest. Consequently, the overall evidence regarding the association of SJL with depression and anxiety remained highly uncertain. Despite this limitation, the study retains scholarly value, as its large sample size and systematic approach may provide meaningful insights and a solid foundation for future research.

Given the key role of SJL in the development of depression and anxiety among adolescents and young people, future research should prioritize longitudinal study designs to more accurately evaluate the causal relationships between SJL with depression and anxiety outcomes. Incorporating objective tools, such as actigraphy and wearable devices, to measure sleep parameters can mitigate recall bias, increase the reliability of the results, and encourage individuals to make adjustments aimed at reducing SJL through real‐time feedback on circadian alignment. Additionally, developing standardized definitions and validated tools for measuring SJL in relation to depression and anxiety outcomes can enhance the reliability, comparability, and overall quality of research in this field. These advancements can enable the development of targeted interventions aimed at mitigating the adverse effects of SJL on depression and anxiety.

Overall, the findings of this review indicate a possible association between SJL and increased odds of depression and anxiety in adolescents and young people. Given the very low certainty of evidence according to the GRADE assessment, these results should be regarded as preliminary and hypothesis‐generating. Further longitudinal and experimental studies that integrate both subjective and objective approaches, including biological markers such as melatonin and cortisol, are needed to elucidate the causal pathways linking SJL with mental health. Integrating multimodal assessments of circadian rhythm and psychological outcomes will be essential for clarifying the underlying physiological mechanisms and for guiding effective preventive and therapeutic strategies.

## 5. Conclusion

In conclusion, the present review provides evidence supporting a possible association between SJL and higher odds of depression and anxiety among adolescents and young people. However, given that the certainty of evidence was rated as very low according to the GRADE assessment, these findings should be interpreted with caution. Future high‐quality longitudinal studies are urgently needed to confirm these associations and to guide interventions addressing sleep–wake misalignment in this population.

## Conflicts of Interest

The authors declare no conflicts of interest.

## Author Contributions

Yi‐An Lu contributed to writing – original draft, visualization, methodology, software, formal analysis, data curation, conceptualization. Pei‐Shan Tsai contributed to writing – review and editing, validation, supervision, conceptualization.

## Funding

This study was supported by the National Science and Technology Council, Taiwan (NSTC 113‐2314‐B‐038‐057‐MY3).

## Supporting Information

Additional supporting information can be found online in the Supporting Information section.

## Supporting information


**Supporting Information** Figure S1: Funnel plots for publication bias. Table S1: Search strategy. Table S2.1: Subgroup analyses of association between SJL and depression stratified by SJL definition. Table S2.2: Subgroup analyses of association between SJL and depression stratified by questionnaire type. Table S2.3: Subgroup analyses of association between SJL and depression stratified by student population. Table S3: Subgroup analyses of association between SJL and anxiety stratified by SJL definition. Table S4: Summary of meta‐regression analysis results. Table S5: Sensitivity analysis of outcomes of interest. Table S6: Summary of GRADE assessments for evidence certainty.

## Data Availability

The datasets generated or analyzed during this study are available from the corresponding author on reasonable request.
