# Supplementary material for: Associations of Social Jetlag With Depression and Anxiety in Adolescents and Young People: A Systematic Review and Meta‐Analysis
Source: Depress Anxiety. 2026 Jan 16;2026:5542425. doi: 10.1155/da/5542425 (PMC12811408; doi:10.1155/da/5542425)
Supplement: Supplementary file 1 — Supporting Information Figure S1: Funnel plots for publication bias. Table S1: Search strategy. Table S2.1: Subgroup analyses of association between SJL and depression stratified by SJL definition. Table S2.2: Subgroup analyses of association between SJL and depression stratified by questionnaire type. Table S2.3: Subgroup analyses of association between SJL and depression stratified by student population. Table S3: Subgroup analyses of association between SJL and anxiety stratified by SJL definition. Table S4: Summary of meta‐regression analysis results. Table S5: Sensitivity analysis of outcomes of interest. Table S6: Summary of GRADE assessments for evidence certainty. [file DA-2026-5542425-s001.docx]

**Depression**


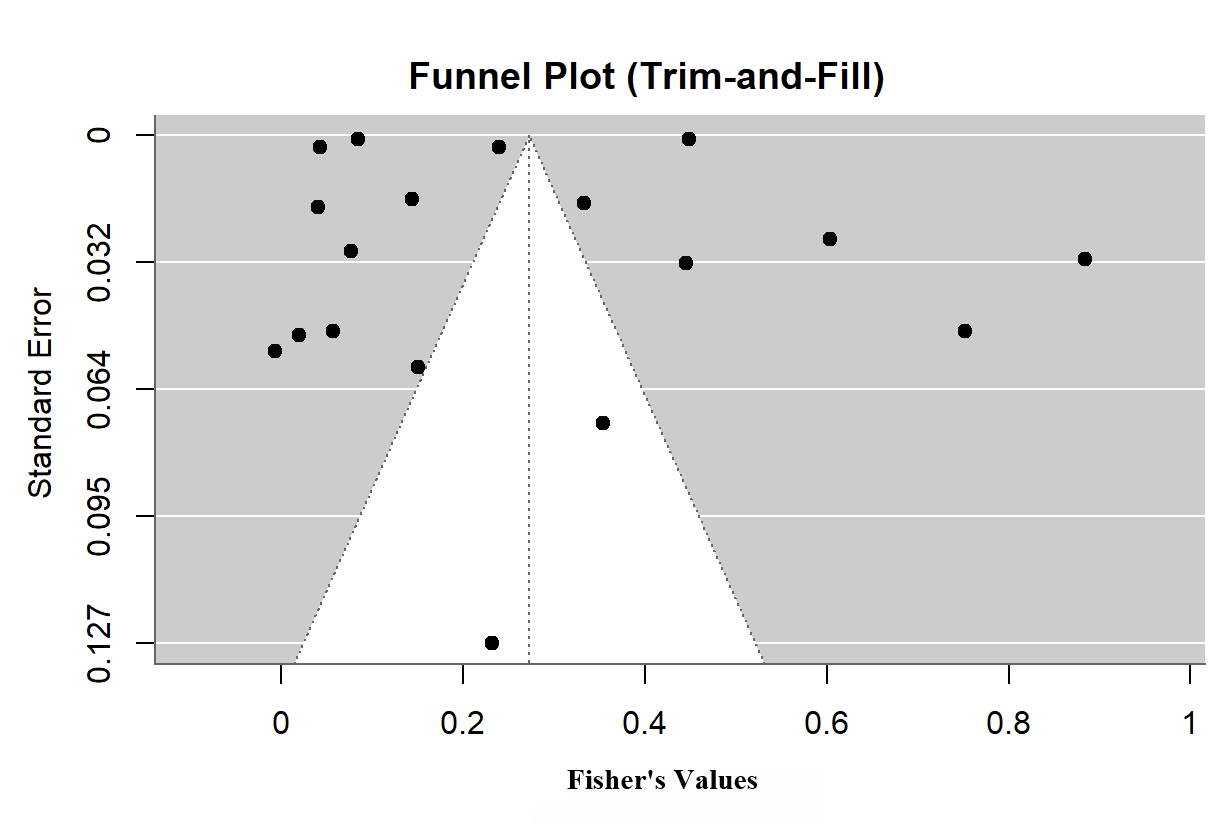


**Supplementary Fig. 1A.** Publication bias.

Egger’s test results: *t* = −0.1410, df = 16, *p* = 0.890

**Anxiety**

**
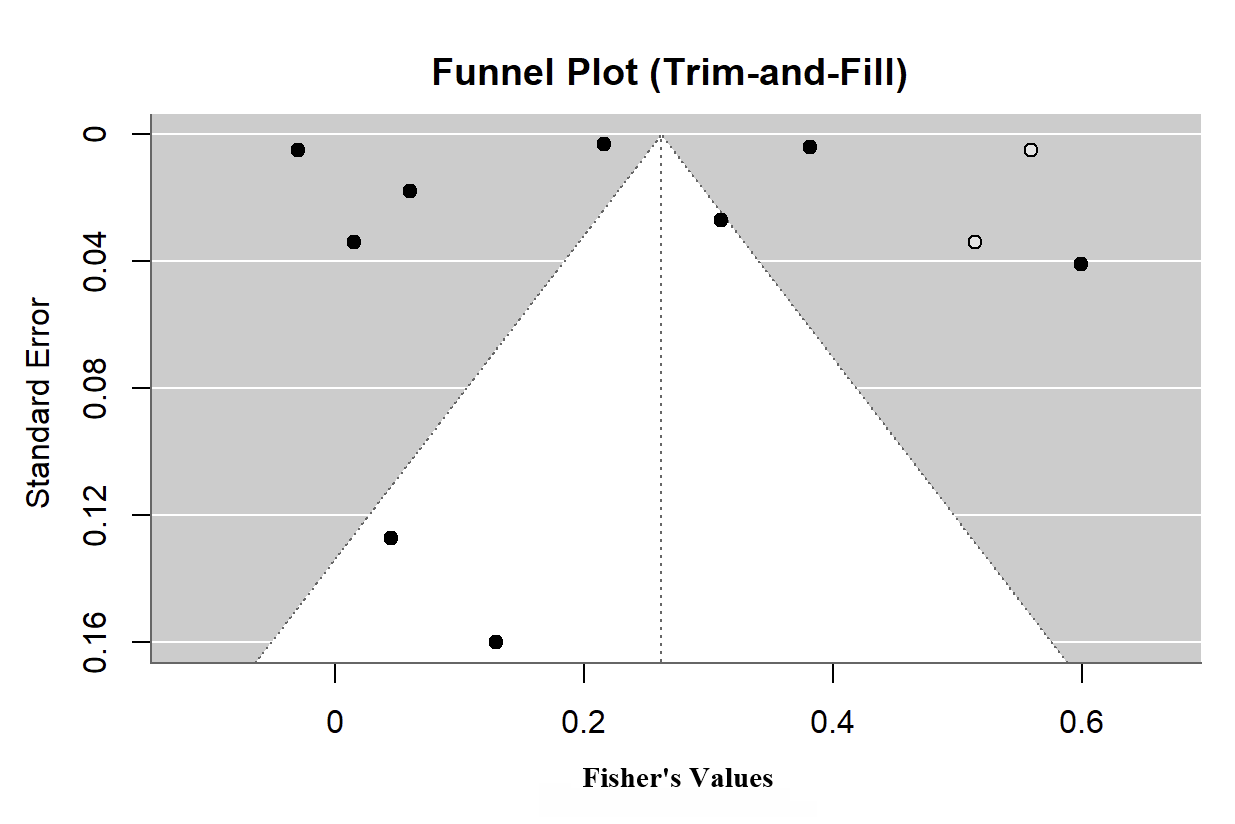
**

**Supplementary Fig. 1B.** Publication bias.

Egger’s test results: *t* = −0.2228, df = 7, *p* = 0.830

**Supplementary Table 1.** Search strategy.

**Database: CINAHL**

|  | Searches | Results |
| --- | --- | --- |
| S1 | TX social jetlag OR TX jetlag OR TX jet lag OR TX jet-lag OR TX sleep debt | 818 |
| S2 | TX anxiety OR TX depression OR TX mental health OR TX psychological | 788,361 |
| S3 | TX adolescent OR TX young people OR TX young adulthood | 1,146,079 |
| S4 | S1 AND S2 AND S3 | 93 |

**Database: Embase**

|  | **Searches** | **Results** |
| --- | --- | --- |
| **#1** | “social jet lag” OR jetlag OR “jet lag” OR “sleep debt” | 5,932 |
| **#2** | anxiety OR depression OR “mental health” OR TX psychological | 2,441,299 |
| **#3** | adolescent OR “young people” OR “young adulthood” | 2,216,156 |
| **#4** | #1 AND #2 AND #3 | 323 |

**Database: PsycINFO**

|  | Searches | Results |
| --- | --- | --- |
| S1 | TX social jet lag OR TX jetlag OR TX jet lag OR TX jet-lag OR TX sleep debt | 1,081 |
| S2 | TX anxiety OR TX depression OR TX mental health OR TX psychological | 2,113,933 |
| S3 | TX adolescent OR TX young people OR TX young adulthood | 1,208,208 |
| S4 | S1 AND S2 AND S3 | 182 |

**Supplementary Table 1.** (*Continued*).

**Database: PubMed**

|  | **Searches** | **Results** |
| --- | --- | --- |
| **#1** | ((((social jet lag) OR (jetlag)) OR (jet lag)) OR (jet-lag)) OR (sleep debt)) | 18,892 |
| **#2** | (((anxiety) OR (depression)) OR (mental health)) OR (psychological) | 1,815,846 |
| **#3** | ((adolescent) OR (young people)) OR (young adulthood) | 13,174,096 |
| **#4** | ((((((social jet lag) OR (jet ag)) OR (jet lag)) OR (jet-lag)) OR (sleep debt)) AND ((((anxiety) OR (depression)) OR (mental health)) OR (psychological))) AND (((adolescent) OR (young people)) OR (young adulthood)) | 1,188 |

**Database: Web of Science**

|  | **Searches** | **Results** |
| --- | --- | --- |
| **#1** | ((((ALL= (social jet lag)) OR ALL=(jetlag)) OR ALL= (jet lag))  OR ALL= (jet-lag)) OR ALL= (sleep debt) | 5,061 |
| **#2** | (((ALL= (anxiety)) OR ALL=(depression)) OR ALL= (mental health))  OR ALL= (psychological) | 2,020,320 |
| **#3** | ((ALL= (adolescent)) OR ALL= (young people)) OR ALL= (young adulthood) | 849,185 |
| **#4** | #1 AND #2 AND #3 | 183 |

**Supplementary Table 2.1** Subgroup analyses of association between SJL and depression stratified by SJL definition.

| Study | Fisher’s *z* | SE | Weight | Fisher’s *z*  IV, random, 95% CI | Fisher’s *z*  IV, random, 95% CI |
| --- | --- | --- | --- | --- | --- |
| **2.1A SJL = 1 and 2 h (SJL < 1 h)** | | | | | 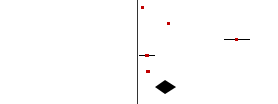  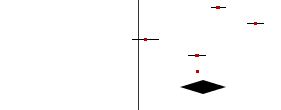  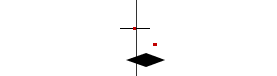  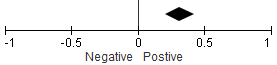 |
| Li et al. (2024), SJL = 1 and 2 h | 0.043 | 0.003 | 8.5% | 0.04 (0.04, 0.05) |  |
| Li et al. (2024), SJLsc = 1 and 2 h | 0.24 | 0.003 | 8.5% | 0.24 (0.23, 0.25) |  |
| Tamura and Okamura (2023)^a^ | 0.752 | 0.049 | 8.0% | 0.75 (0.66, 0.85) |  |
| Tamura and Okamura (2023)^b^ | 0.077 | 0.029 | 8.3% | 0.08 (0.02, 0.13) |  |
| Zhang et al. (2023) | 0.085 | 0.001 | 8.5% | 0.09 (0.08, 0.09) |  |
| Subtotal (95% CI) |  |  | 42.0% | 0.22 (0.14, 0.30) |  |
| Heterogeneity: τ^2^ = 0.01, χ^2^ = 2,916.44, df = 4 (*p* < 0.00001), *I*^2^ = 100% | | | | |  |
| Test for overall effect: *Z* = 5.38 (*p* < 0.00001) | | | | |  |
| **2.1B SJL ≥ 2 h (SJL < 1 h)** | | | | |  |
| Li et al. (2024), SJL ≥ 2 h | 0.604 | 0.026 | 8.4% | 0.60 (0.55, 0.65) |  |
| Li et al. (2024), SJLsc ≥ 2 h | 0.884 | 0.031 | 8.3% | 0.88 (0.82, 0.94) |  |
| Tamura and Okamura (2023)^a^ | 0.057 | 0.049 | 8.0% | 0.06 (−0.04, 0.15) |  |
| Tamura and Okamura (2023)^b^ | 0.445 | 0.032 | 8.3% | 0.45 (0.38, 0.51) |  |
| Zhang et al. (2023) | 0.449 | 0.001 | 8.5% | 0.45 (0.45, 0.45) |  |
| Subtotal (95% CI) |  |  | 41.6% | 0.49 (0.32, 0.66) |  |
| Heterogeneity: τ^2^ = 0.04, χ^2^ = 296.20, df = 4 (*p* < 0.00001), *I*^2^ = 99% | | | | |  |
| Test for overall effect: *Z* = 5.60 (*p <* 0.00001) | | | | |  |
| **2.1C** **SJL ≥ 2 h (SJL < 2 h)** |  |  |  |  |  |
| de Souza et al. (2014) | −0.007 | 0.054 | 7.9% | −0.01 (−0.11, 0.10) |  |
| Wong et al. (2024) | 0.144 | 0.016 | 8.5% | 0.14 (0.11, 0.18) |  |
| Subtotal (95% CI) |  |  | 16.4% | 0.08 (−0.07, 0.22) |  |
| Heterogeneity: τ^2^ = 0.01, χ^2^ = 7.82, df = 1 (*p =* 0.005), *I*^2^ = 87% | | | | |  |
| Test for overall effect: *Z* = 1.04 (*p =* 0.30) | | | | |  |
|  |  |  |  |  |  |
| Total (95% CI) |  |  | 100% | 0.31 (0.21, 0.42) |  |
| Heterogeneity: τ^2^ = 0.04, χ^2^ = 80,863.57, df = 11 (*p* < 0.00001), *I*^2^ = 100% | | | | |  |
| Test for overall effect: *Z* = 5.72 (*p* < 0.00001) | | | | |  |
| Test for subgroup differences: χ^2^ = 13.8, df = 2 (*p* = 0.001), *I*^2^ = 84.7% | | | | |  |

SJL = social jetlag; SJLsc = sleep-corrected social jetlag.

**Supplementary Table 2.2** Subgroup analyses of association between SJL and depression stratified by questionnaire type.

| Study | Fisher’s *z* | SE | Weight | Fisher’s *z*  IV, random, 95% CI | Fisher’s *z*  IV, random, 95% CI |
| --- | --- | --- | --- | --- | --- |
| **2.2A Beck Depression Inventory** | | | | | 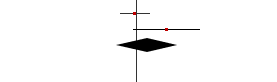  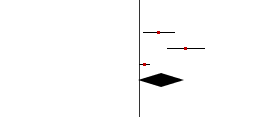  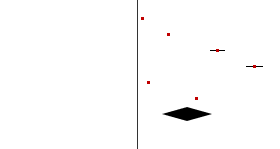  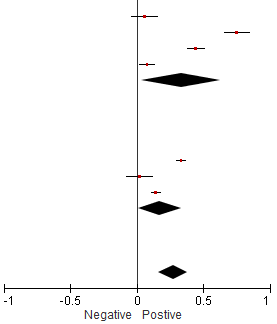 |
| de Souza et al. 2014), SJL ≥ 2 h | −0.007 | 0.054 | 5.5% | −0.01 (−0.11, 0.10) |  |
| Magnusdottir et al. (2024) | 0.232 | 0.127 | 4.4% | 0.23 (−0.02, 0.48) |  |
| Subtotal (95% CI) |  |  | 9.9% | 0.08 (−0.14, 0.31) |  |
| Heterogeneity: τ^2^ = 0.02, χ^2^ = 3.00, df = 1 (*p* < 0.08), *I*^2^ = 67% | | | | |  |
| Test for overall effect: *Z* = 0.73 (*p* = 0.47) | | | | |  |
| **2.2B Center for Epidemiological Studies Depression Scale** | | | | |  |
| Jang et al. (2021) | 0.151 | 0.058 | 5.4% | 0.15 (1.04, 0.26) |  |
| Jang and Lee (2023) | 0.354 | 0.072 | 5.3% | 0.35 (0.21, 0.50) |  |
| Mathew et al. (2019)^a^ | 0.04 | 0.018 | 5.7% | 0.04 (0.00, 0.08) |  |
| Subtotal (95% CI) |  |  | 16.4% | 0.17 (−0.00, 0.34) |  |
| Heterogeneity: τ^2^ = 0.02, χ^2^ = 20.23, df = 2 (*p* < 0.0001), *I*^2^ = 90% | | | | |  |
| Test for overall effect: *Z* = 1.93 (*p* = 0.05) | | | | |  |
| **2.2C Patient Health Questionnaire** | | | | |  |
| Li et al. (2024), SJL = 1 and 2 h | 0.043 | 0.003 | 5.8% | 0.04 (0.04, 0.05) |  |
| Li et al. (2024), SJLsc = 1 and 2 h | 0.24 | 0.003 | 5.8% | 0.24 (0.23, 0.25) |  |
| Li et al. (2024), SJL ≥ 2 h | 0.604 | 0.026 | 5.7% | 0.60 (0.55, 0.65) |  |
| Li et al. (2024), SJLsc ≥ 2 h | 0.884 | 0.031 | 5.7% | 0.88 (0.82, 0.94) |  |
| Zhang et al. (2023), SJL = 1 and 2 h | 0.085 | 0.001 | 5.8% | 0.09 (0.08, 0.09) |  |
| Zhang et al. (2023), SJL ≥ 2 h | 0.449 | 0.001 | 5.8% | 0.45 (0.45, 0.45) |  |
| Subtotal (95% CI) |  |  | 34.4% | 0.38 (0.19, 0.57) |  |
| Heterogeneity: τ^2^ = 0.05, χ^2^ = 72,146.65, df = 5 (*p* < 0.00001), *I*^2^ = 100% | | | | |  |
| Test for overall effect: *Z* = 3.99 (*p <* 0.00001) | | | | |  |
| **2.2D** **Depression Self-Rating Scale for Children** | | | | |  |
| Tamura and Okamura (2023),^a^ SJL = 1 and 2 h | 0.057 | 0.049 | 5.5% | 0.06 (−0.04, 0.15) |  |
| Tamura and Okamura (2023),^a^ SJL ≥ 2 h | 0.752 | 0.049 | 5.5% | 0.75 (0.66, 0.85) |  |
| Tamura and Okamura (2023),^b^ SJL = 1 and 2 h | 0.445 | 0.032 | 5.7% | 0.45 (0.38, 0.51) |  |
| Tamura and Okamura (2023),^b^ SJL ≥ 2 h | 0.077 | 0.029 | 5.7% | 0.08 (0.02, 0.13) |  |
| Subtotal (95% CI) |  |  | 22.4% | 0.33 (0.04, 0.63) |  |
| Heterogeneity: τ^2^ = 0.09, χ^2^ = 188.90, df = 3 (*p* < 0.00001), *I*^2^ = 98% | | | | |  |
| Test for overall effect: *Z* = 2.21 (*p* = 0.03) | | | | |  |
| **2.2E Other questionnaires** | | | | |  |
| Borisenkov et al. (2015) | 0.333 | 0.017 | 5.7% | 0.33 (0.30, 0.37) |  |
| Sheaves et al. (2016) | 0.02 | 0.05 | 5.5% | 0.02 (−0.08, 0.12) |  |
| Wong et al. (2024), SJL ≥ 2 h | 0.144 | 0.016 | 5.7% | 0.14 (0.11, 0.18) |  |
| Subtotal (95% CI) |  |  | 17.0% | 0.17 (0.01, 0.33) |  |
| Heterogeneity: τ^2^ = 0.02, χ^2^ = 82.72, df = 2 (*p* < 0.00001), *I*^2^ = 98% | | | | |  |
| Test for overall effect: *Z* = 4.59 (*p* = 0.04) | | | | |  |
| Total (95% CI) |  |  | 100% | 0.27 (0.16, 0.38) |  |
| Heterogeneity: τ^2^ = 0.05, χ^2^ = 72,600.98, df = 17 (*p* < 0.00001), *I*^2^ = 100% | | | | |  |
| Test for overall effect: *Z* = 4.88 (*p* < 0.00001) | | | | |  |
| Test for subgroup differences: χ^2^ = 5.44, df = 4 (*p* = 0.25), *I*^2^ = 26.4% | | | | |  |

SJL = social jetlag; SJLsc = sleep-corrected social jet lag.

**Supplementary Table 2.3** Subgroup analyses of association between SJL and depression stratified by student population.

| Study | Fisher’s *z* | SE | Weight | Fisher’s *z*  IV, random, 95% CI | | Fisher’s *z*  IV, random, 95% CI | |
| --- | --- | --- | --- | --- | --- | --- | --- |
| **2.3A College students (aged 19–23 years)** | | | | | | | 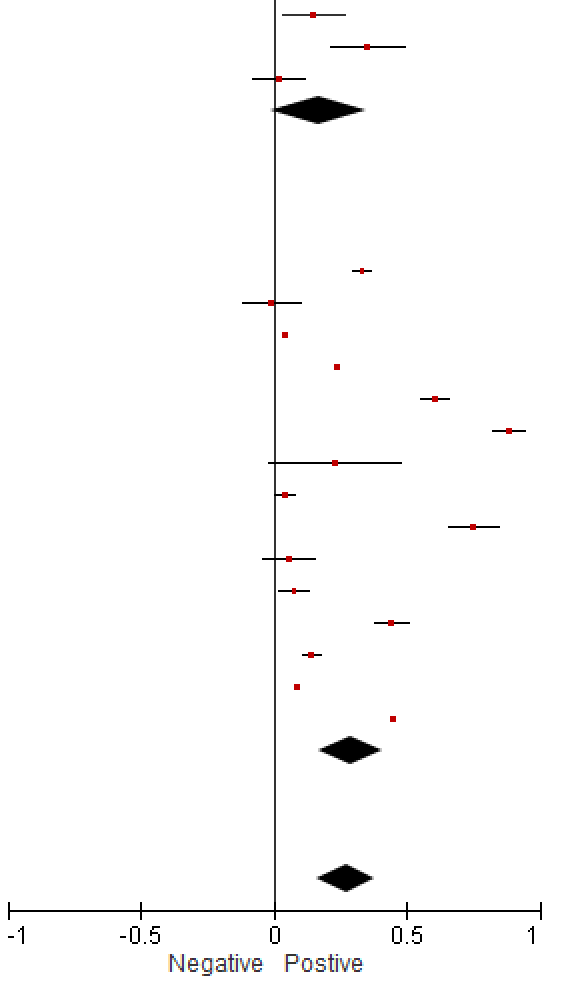 |
| Jang et al. (2021) | 0.151 | 0.058 | 5.4% | | 0.15 (1.04, 0.26) | |  |
| Jang and Lee (2023) | 0.354 | 0.072 | 5.3% | | 0.35 (0.21, 0.50) | |  |
| Sheaves et al. (2016) | 0.02 | 0.05 | 5.5% | | 0.02 (−0.08, 0.12) | |  |
| Subtotal (95% CI) |  |  | 16.2% | | 0.17 (−0.01, 0.35) | |  |
| Heterogeneity: τ^2^ = 0.02, χ^2^ = 14.62, df = 2 (*p =* 0.0007), *I*^2^ = 86% | | | | | | |  |
| Test for overall effect: *Z* = 1.82 (*p* = 0.07) | | | | | | |  |
| **2.3B Students (mixed)** | | | | | | |  |
| Borisenkov et al. (2015) | 0.333 | 0.017 | 5.7% | | 0.33 (0.30, 0.37) | |  |
| De Souza et al. (2014), SJL ≥ 2 h | −0.007 | 0.054 | 5.5% | | −0.01 (−0.11, 0.10) | |  |
| Li et al. (2024), SJL = 1 and 2 h | 0.043 | 0.003 | 5.8% | | 0.04 (0.04, 0.05) | |  |
| Li et al. (2024), SJLsc = 1 and 2 h | 0.24 | 0.003 | 5.8% | | 0.24 (0.23, 0.25) | |  |
| Li et al. (2024), SJL ≥ 2 h | 0.604 | 0.026 | 5.7% | | 0.60 (0.55, 0.65) | |  |
| Li et al. (2024), SJLsc ≥ 2 h | 0.884 | 0.031 | 5.7% | | 0.88 (0.82, 0.94) | |  |
| Magnusdottir et al. (2024) | 0.232 | 0.127 | 4.4% | | 0.23 (−0.02, 0.48) | |  |
| Mathew et al. (2019)^a^ | 0.04 | 0.018 | 5.7% | | 0.04 (0.00, 0.08) | |  |
| Tamura and Okamura (2023),^a^ SJL = 1 and 2 h | 0.752 | 0.049 | 5.5% | | 0.75 (0.66, 0.85) | |  |
| Tamura and Okamura (2023),^a^ SJL ≥ 2 h | 0.057 | 0.049 | 5.5% | | 0.06 (−0.04, 0.15) | |  |
| Tamura and Okamura (2023),^b^ SJL = 1 and 2 h | 0.077 | 0.029 | 5.7% | | 0.08 (0.02, 0.13) | |  |
| Tamura and Okamura (2023),^b^ SJL ≥ 2 h | 0.455 | 0.032 | 5.7% | | 0.45 (0.38, 0.51) | |  |
| Wong et al. (2024), SJL ≥ 2 h | 0.144 | 0.016 | 5.7% | | 0.14 (0.11, 0.18) | |  |
| Zhang et al. (2023), SJL = 1 and 2 h | 0.085 | 0.001 | 5.8% | | 0.09 (0.08, 0.09) | |  |
| Zhang et al. (2023), SJL ≥ 2 h | 0.449 | 0.001 | 5.8% | | 0.45 (0.45, 0.45) | |  |
| Subtotal (95% CI) |  |  | 83.8% | | 0.37 (0.21, 0.53) | |  |
| Heterogeneity: τ^2^ = 0.05, χ^2^ = 72,573.87, df = 17 (*p* < 0.00001), *I*^2^ = 100% | | | | | | |  |
| Test for overall effect: *Z* = 4.78 (*p* = 0.00001) | | | | | | |  |
| Total (95% CI) |  |  | 100% | | 0.27 (0.16, 0.38) | |  |
| Heterogeneity: τ^2^ = 0.05, χ^2^ = 72,600.98, df = 17 (*p* < 0.00001), *I*^2^ = 100% | | | | | | |  |
| Test for overall effect: *Z* = 4.88 (*p* = 0.00001) | | | | | | |  |
| Test for subgroup differences: χ^2^ = 1.23, df = 1 (*p* = 0.27), *I*^2^ = 18.8% | | | | | | |  |

SJL = social jet lag; SJLsc = sleep-corrected social jet lag.

**Supplementary Table 3.** Subgroup analyses of association between SJL and anxiety stratified by SJL definition.

| Study | Fisher’s *z* | SE | Weight | Fisher’s *z*  IV, random, 95% CI | | Fisher’s *z*  IV, random, 95% CI |
| --- | --- | --- | --- | --- | --- | --- |
| **3A. SJL = 1 and 2 h (SJL < 1 h)** | | | | | | 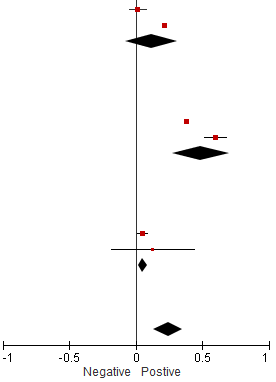 |
| Li et al. (2024), SJL = 1 and 2 h | 0.015 | 0.034 | 17.9% | | 0.01 (−0.05, 0.08) |  |
| Li et al. (2024), SJLsc = 1 and 2 h | 0.216 | 0.003 | 19.3% | | 0.22 (0.21, 0.22) |  |
| Subtotal (95% CI) |  |  | 37.2% | | 0.12 (−0.08, 0.32) |  |
| Heterogeneity: τ^2^ = 0.02, χ^2^ = 34.68, df = 1 (*p* < 0.00001), *I*^2^ = 97% | | | | | |  |
| Test for overall effect: *Z* = 1.18 (*p* = 0.24) | | | | | |  |
| **3B. SJL ≥ 2 h (SJL < 1 h)** | | | | | |  |
| Li et al. (2024), SJL ≥ 2 h | 0.381 | 0.004 | 19.2% | | 0.38 (0.37, 0.39) |  |
| Li et al. (2024), SJLsc ≥ 2 h | 0.599 | 0.041 | 17.4% | | 0.56 (0.48, 0.64) |  |
| Subtotal (95% CI) |  |  | 36.6% | | 0.49 (0.27, 0.70) |  |
| Heterogeneity: τ^2^ = 0.02, χ^2^ = 28.00, df = 1 (*p* < 0.00001), *I*^2^ = 96% | | | | | |  |
| Test for overall effect: *Z* = 4.46 (*p* < 0.00001) | | | | | |  |
| **3C. SJL ≥ 2 h (SJL < 2 h)** |  |  |  | |  |  |
| Mathew et al. (2019)^b^ | 0.05 | 0.018 | 18.9% | | 0.05 (0.01, 0.09) |  |
| Wong et al. (2024) | 0.129 | 0.16 | 7.3% | | 0.13 (−0.18, 0.44) |  |
| Subtotal (95% CI) |  |  |  | | 0.05 (0.02, 0.09) |  |
| Heterogeneity: τ^2^ = 0.02, χ^2^ = 30.52, df = 2 (*p* < 0.0001), *I*^2^ = 93% | | | | | |  |
| Test for overall effect: *Z* = 4.28 (*p* < 0.0001) | | | | | |  |
|  |  |  |  | |  |  |
| Total (95% CI) |  |  | 100% | | 0.24 (0.13, 0.35) |  |
| Heterogeneity: τ^2^ = 0.02, χ^2^ = 1,364.57, df = 5 (*p* < 0.00001), *I*^2^ = 100% | | | | | |  |
| Test for overall effect: *Z* = 4.38 (*p* < 0.0001) | | | | | |  |
| Test for subgroup differences: χ^2^ = 15.84, df = 2 (*p* = 0.0004), *I*^2^ = 87.4% | | | | | |  |

SJL = social jet lag; SJLsc = sleep-corrected social jet lag.

**Supplementary Table 4.** Summary of meta-regression analysis results.

| Outcomes | No. of studies | Coefficient | Standard error | 95% CI | *p* value |
| --- | --- | --- | --- | --- | --- |
| **Depression** |  |  |  |  |  |
| Age | 12 | 0.0428 | 0.291 | −0.142, 0.997 | 0.141 |
| Female | 12 | 0.220 | 0.234 | −0.238, 0.679 | 0.347 |
|  |  |  |  |  |  |
| **Anxiety** |  |  |  |  |  |
| Age | 6 | 0.933 | 0.382 | 0.183, 1.684 | 0.015^*^ |
| Female | 6 | 1.119 | 0.698 | −0.250, 2.488 | 0.109 |

Note: ^*^*p* < 0.05.

**Supplementary Table 5.** Sensitivity analysis of outcomes of interest.

**Depression**

| Study | Fisher’s *z* | SE | Weight | Fisher’s *z*  IV, random, 95% CI | Fisher’s *z*  IV, random, 95% CI |
| --- | --- | --- | --- | --- | --- |
| Jang et al. (2021) | 0.151 | 0.058 | 7.4% | 0.15 (0.04, 0.26) | 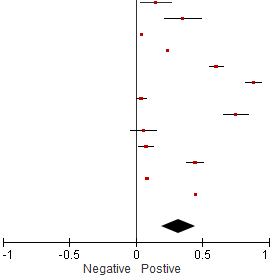 |
| Jang and Lee (2023) | 0.354 | 0.072 | 7.2% | 0.35 (0.21, 0.50) |  |
| Li et al. (2024), SJL = 1 and 2 h | 0.043 | 0.003 | 7.9% | 0.04 (0.04, 0.05) |  |
| Li et al. (2024), SJLsc = 1 and 2 h | 0.24 | 0.003 | 7.9% | 0.24 (0.23, 0.25) |  |
| Li et al. (2024), SJL ≥ 2 h | 0.604 | 0.026 | 7.8% | 0.60 (0.55, 0.66) |  |
| Li et al. (2024), SJLsc ≥ 2 h | 0.884 | 0.031 | 7.7% | 0.88 (0.82, 0.94) |  |
| Mathew et al. (2019)^a^ | 0.04 | 0.018 | 7.8% | 0.04 (0.00, 0.08) |  |
| Tamura and Okamura (2023),^a^ SJL = 1 and 2 h | 0.752 | 0.049 | 7.5% | 0.75 (0.66, 0.85) |  |
| Tamura and Okamura (2023),^a^ SJL ≥ 2 h | 0.057 | 0.049 | 7.5% | 0.06 (−0.04, 0.15) |  |
| Tamura and Okamura (2023),^b^ SJL = 1 and 2 h | 0.077 | 0.029 | 7.8% | 0.08 (0.02, 0.13) |  |
| Tamura and Okamura (2023),^b^ SJL ≥ 2 h | 0.445 | 0.032 | 7.7% | 0.45 (0.38, 0.51) |  |
| Zhang et al. (2023), SJL = 1 and 2 h | 0.085 | 0.001 | 7.9% | 0.09 (0.08, 0.09) |  |
| Zhang et al. (2023), SJL ≥ 2 h | 0.449 | 0.001 | 7.9% | 0.45 (0.45, 0.45) |  |
|  |  |  |  |  |  |
| Total (95% CI) |  |  | 100% | 0.32 (0.19, 0.45) |  |
| Heterogeneity: τ^2^ = 0.05, χ^2^ = 72,486.39, df = 12 (*p* < 0.00001), *I*^2^ = 100% | | | | |  |
| Test for overall effect: *Z* = 4.89 (*p* < 0.00001) | | | | |  |

SJL = social jet lag; SJLsc = sleep-corrected social jet lag.

**Supplementary Table 5.** (*Continued*).

**Anxiety**

| Study | Fisher’s *z* | SE | Weight | Fisher’s *z*  IV, random, 95% CI | Fisher’s *z*  IV, random, 95% CI |
| --- | --- | --- | --- | --- | --- |
| Li et al. (2024), SJL = 1 and 2 h | 0.015 | 0.034 | 19.4% | 0.01 (−0.05, 0.08) | 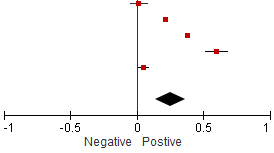 |
| Li et al. (2024), SJLsc = 1 and 2 h | 0.216 | 0.003 | 20.8% | 0.22 (0.21, 0.22) |  |
| Li et al. (2024), SJL ≥ 2 h | 0.381 | 0.004 | 20.8% | 0.38 (0.37, 0.39) |  |
| Li et al. (2024), SJLsc ≥ 2 h | 0.599 | 0.041 | 18.8% | 0.60 (0.52, 0.68) |  |
| Mathew et al. (2019)^b^ | 0.05 | 0.018 | 20.4% | 0.05 (0.01, 0.09) |  |
|  |  |  |  |  |  |
| Total (95% CI) |  |  | 100% | 0.25 (0.14, 0.36) |  |
| Heterogeneity: τ^2^ = 0.02, χ^2^ = 1,363.78, df = 8 (*p* < 0.00001), *I*^2^ = 100% | | | | |  |
| Test for overall effect: *Z* = 4.37 (*p* < 0.0001) | | | | |  |

SJL = social jet lag; SJLsc = sleep-corrected social jet lag.

**Supplementary Table 6.** Summary of GRADE assessments for evidence certainty.

| Quality of evidence | | | | | | | | No. of participants | Effect | | | Certainty of evidence |
| --- | --- | --- | --- | --- | --- | --- | --- | --- | --- | --- | --- | --- |
| No. of studies | Study design | Risk of bias | Inconsistency | Indirectness | Imprecision | Publication bias | Other considerations |  | Effect size  (Fisher’s *z*) | 95% CI  (lower limit, upper limit) | *I*^2^ |  |
| **Depression** | | | | | | | | | | | | |
| 12 | Cross-sectional or cohort study | Serious | Serious | Serious | Not serious | Not detected | None | 158,623 | 0.27 | 0.16, 0.38 | 99.98% | ⨁◯◯◯  Very low |
| **Anxiety** | | | | | | | | | | | | |
| 6 | Cross-sectional or cohort study | Serious | Serious | Serious | Serious | Not detected | None | 117,353 | 0.21 | 0.21, 0.29 | 99.86% | ⨁◯◯◯  Very low |
